# A Solid Dispersion of Quercetin Shows Enhanced Nrf2 Activation and Protective Effects against Oxidative Injury in a Mouse Model of Dry Age-Related Macular Degeneration

**DOI:** 10.1155/2019/1479571

**Published:** 2019-11-07

**Authors:** Yan Shao, Haitao Yu, Yan Yang, Min Li, Li Hang, Xinrong Xu

**Affiliations:** ^1^Department of Ophthalmology, The Affiliated Hospital of Nanjing University of Chinese Medicine, Nanjing 210029, China; ^2^School of Pharmacy, Nanjing University of Chinese Medicine, Nanjing 210023, China; ^3^Department of Ophthalmology, The Second Affiliated Hospital of Nanjing University of Chinese Medicine, Nanjing 210017, China; ^4^Department of Ophthalmology, Liyang Branch of the Affiliated Hospital of Nanjing University of Chinese Medicine, Liyang 213300, China

## Abstract

Age-related macular degeneration (AMD) represents a major reason for blindness in the elderly population. Oxidative stress is a predominant factor in the pathology of AMD. We previously evaluated the effects of phospholipid complex of quercetin (Q-PC) on oxidative injury in ARPE-19 cells, but the underlying mechanisms are not fully understood. Herein, the solid dispersion of quercetin-PC (Q-SD) was prepared with solubility being 235.54 *μ*g/mL in water and 2.3×10^4^ *μ*g/mL in chloroform, which were significantly higher than that of quercetin (QT) and Q-PC. Q-SD also exhibited a considerably higher dissolution rate than QT and Q-PC. Additionally, Q-SD had Cmax of 4.143 *μ*g/mL and AUC of 12.015 *μ*g·h/mL in rats, suggesting better bioavailability than QT and Q-PC. Then, a mouse model of dry AMD (Nrf2 wild-type (WT) and Nrf2 knockout (KO)) was established for evaluating the effects of Q-SD in vivo. Q-SD more potently reduced retinal pigment epithelium sediments and Bruch's membrane thickness than QT and Q-PC at 200 mg/kg in Nrf2 WT mice and did not work in Nrf2 KO mice at the same dosage. Additionally, Q-SD significantly decreased ROS and MDA contents and restored SOD, GSH-PX, and CAT activities of serum and retinal tissues in Nrf2 WT mice, but not in Nrf2 KO mice. Furthermore, Q-SD more potently increased Nrf2 mRNA expression and stimulated its nuclear translocation in retinal tissues of Nrf2 WT mice. Q-SD significantly increased the expression of Nrf2 target genes HO-1, HQO-1, and GCL of retinal tissues in Nrf2 WT mice, not in Nrf2 KO mice. Altogether, Q-SD had improved physicochemical and pharmacokinetic properties compared to QT and Q-PC and exhibited more potent protective effects on retina oxidative injury in vivo. These effects were associated with activation of Nrf2 signaling and upregulation of antioxidant enzymes.

## 1. Introduction

Age-related macular degeneration (AMD) is a leading irreversible blindness in elderly people all over the world. This disease can be generally divided into two categories, namely, dry AMD and wet AMD. The former is characterized by choroidal capillary atrophy, drusen, and retinal pigment epithelium (RPE) atrophy, and geographic atrophy of the macular region commonly occurs in its advanced stage, resulting in decreased visual acuity and even wet AMD. The wet AMD is primarily featured by choroidal neovascularization (CNV), leading to retinal exudation and hemorrhage and eventually serious impairment of vision [1, 2]. Recently, much progress has been made in the management of wet AMD due to the application of anti-VEGF (vascular endothelial growth factor) agents in clinical practice [3, 4]. However, the underlying mechanism of dry AMD pathology remains largely unknown, and thus, there are no effective therapeutic options for dry AMD.

A large number of studies have highlighted a critical role for oxidative stress in the pathology of dry AMD [5]. Oxidative stress reflects an imbalance between the systemic manifestation of reactive oxygen species (ROS) and a biological system's ability to readily detoxify the reactive intermediates or to repair the resulting damage [6]. Long-term light exposure can stimulate lipofuscin-mediated oxidative stress in the macula and produce a large amount of superoxide anion, singlet oxygen, and hydrogen peroxide, leading to RPE cell injuries and aggravating the pathogenesis of dry AMD [7]. The nuclear factor erythroid 2-related factor 2 (Nrf2) is a basic leucine zipper protein that regulates the expression of antioxidant proteins that protect against oxidative damage triggered by injury and inflammation, thus serving as a pivotal regulator of redox system in mammal cells [8]. Under normal or unstressed conditions, Nrf2 is kept in the cytoplasm by a cluster of proteins that degrade it quickly. Under oxidative stress, however, Nrf2 is not degraded but instead travels to the nucleus where it binds to antioxidant response elements (AREs) and initiates transcription of antioxidant genes and their proteins [9]. Activation of Nrf2 can induce the expression of many antioxidases and phase II metabolizing enzymes, heme oxygenase-1 (HO-1), quinone oxidoreductase-1 (NQO-1), glutathione-S-transferase, glutamate cysteine ligase (GCL), superoxide dismutase (SOD), catalase (CAT), and glutathione peroxidase (GSH-PX) [10]. These enzymes can quickly scavenge ROS and protect the body from injuries caused by active substances or toxic substances. In addition, they can also regulate cell proliferation and death and enhance the ability to scavenge ROS, thus maintaining intracellular redox balance and reduce oxidative damage.

Quercetin (QT) is a naturally occurring flavonoid compound. Increasing investigations have demonstrated that QT has antioxidant, ROS-scavenging, anti-inflammatory, and antitumor effects [11]. However, QT has the disadvantages of poor water and fat solubility and low bioavailability [12]. Our previous studies showed that the phospholipid complex of quercetin (Q-PC) had a threefold increase in water solubility and 734-fold increase in fat solubility and exhibited stronger protective effects than QT against oxidative injury in ARPE-19 cells by increasing cell viability and reducing apoptosis [13]. Moreover, Q-PC was also found to have stronger protective effects against liver oxidative injury than quercetin in rats [14]. Although formation of phospholipid complex significantly increased the solubility of QT, the hydrophobicity of phospholipid complex resulted in poor dispersity of QT [15]. Therefore, the phospholipid complex had limited increase in bioavailability. Studies have indicated that preparation of solid dispersion can significantly increase the solubility and dissolution rate of drugs, which can further enhance the bioavailability of drugs [16, 17]. In the current study, we successfully prepared the solid dispersion of quercetin phospholipid complex (Q-SD), determined its bioavailability in rats, and evaluated its protective effects on retinal injury caused by oxidative stress in Nrf2 wild-type and Nrf2 knockout mice.

## 2. Materials and Methods

### 2.1. Chemicals, Reagents, and Antibodies

Quercetin was obtained from Sigma Chemical (St. Louis, MO, USA). Reagent-grade soy lecithin (purity > 97%) was purchased from Shanghai Guyan Industry Co., Ltd. (Shanghai, China). Polyvinylpyrrolidone (PVP K30) was purchased from J&K Chemical Ltd. (Shanghai, China). Hydroquinone was obtained from Alfa Aesar (Heysham, Lancashire, UK). The primary antibodies used in Western blot analyses against Nrf2, HO-1, NQO-1, GCL, GAPDH, and Lamin B, and the secondary antibody Goat Anti-Rabbit IgG/HRP were all obtained from Abcam (Cambridge, UK).

### 2.2. Perpetration of Q-SD

We used the solvent method to prepare Q-SD. Briefly, quercetin and soy lecithin (mass ratio 1 : 1) were dissolved in ethanol of adequate volume stirred for 1 h. Decompression distillation was carried out to remove ethanol. After 12 h vacuum drying, the resulting precipitates were grinded and sieved through 100 mesh sieve, yielding Q-PC stored in a desiccator for subsequent experiments. Next, the prepared Q-PC and PVP K30 at a mass ratio of 1 : 3 were dissolved in absolute ethanol of adequate volume with full mixing. This ratio was determined by orthogonal tests via preliminary experiments. After the mixture was evaporated in vacuo at 50°C, the Q-SD was obtained for experiments.

We then determined the standard curve of Q-SD. The chromatographic conditions were a Waters 600 C_l8_ column, methanol 0.4% H_3_PO_4_ solution (*v*/*v* 50 : 50) as the mobile phase, 370 nm wavelength, 1.0 mL/min flow velocity, 30°C column temperature, and 20 *μ*L sample size. The control solution was prepared according to following procedures. QT of 10 mg was dissolved in ethanol and volumed in a 50 mL volumetric flask, and thereby the QT control solution at a concentration of 200 mg/L was obtained. Anhydrous ethanol was used to dilute to the control solution to yield a series of solutions at 10, 20, 40, 60, 80, and 100 mg/L. Each solution of 20 *μ*L was injected into a high-performance liquid chromatograph (HPLC, Waters 600, USA) for measurement of the peak area. Concentrations of QT ([C]) were marked at the horizontal ordinate and peak areas (A) at the longitudinal coordinate giving the standard curve equation A = 4986.6 × [C] − 10.43219 (R = 0.9998), linear range 2.06 − 93.7 *μ*g/mL.

### 2.3. Determination of Q-SD Solubility

QT, Q-PC, and Q-SD (all containing 20 mg pure quercetin) were precisely weighed and added into a 100 mL conical flask, and then distilled water/chloroform of 20 mL was added. The solutions were incubated in a 25°C thermostatic oscillator for 6 h. Each sample of 5 mL was filtered through 0.45 *μ*m microporous membrane. The successive filtrate of 10 *μ*L was subjected to HPLC analyses. Control solution of 10 *μ*L at 40 mg/L was spontaneously analyzed by HPLC. The equilibrium solubility of the three kinds of samples in water and chloroform was measured, respectively, based on the peak area.

### 2.4. Determination of Dissolution Rate of Q-SD

QT, Q-PC at a mass ratio of 1 : 1, and Q-SD at a mass ratio of 1 : 1 : 3 (all containing 20 mg pure quercetin) were evenly dispersed in 200 mL distilled water at 37°C and centrifuged at 100 r/min. Each sample of 2 mL was collected at the time points of 10, 20, 30, 40, 50, and 60 min and was subjected to filtration. The successive filtrate of 1 mL was volumed in a 10 mL volumetric flask with distilled water. HPLC analyses were used, and mass concentrations were calculated through introducing the peak area into the standard curve equation followed by calculation of the cumulative dissolution percentage.

### 2.5. Determination of Serum Concentrations of Q-SD

Eighteen Sprague Dawley rats obtained from the Model Animal Research Centre of Nanjing University (Nanjing, China) were randomly divided into three groups, namely, the QT treatment group, Q-PC treatment group, and Q-SD treatment group (*n* = 6). All rats in the three groups were intragastric administrated with corresponding drugs containing pure QT at 100 mg/kg. Blood samples were collected from orbit at time points of 0.25, 0.5, 0.75, 1, 1.5, 2, 4, and 8 h in each rat. After adding heparin, blood samples were centrifuged at 3000 r/min for 15 min, and the supernatants were collected for examinations. Next, each sample of 0.2 mL was added into a 10 mL centrifuge tube and incubated with 0.2 mL 25% hydrochloric acid in water bath at 80°C for 1 h. Ethyl acetate of 1 mL was added to each sample followed by vortex for 3 min and centrifugation at 3000 r/min for 10 min. The upper organic phase was collected with addition of 1 mL ethyl acetate, and the abovementioned procedures were repeated. The two upper organic phases were combined together and subjected to nitrogen drying in 50°C water bath followed by dissolution with 200 *μ*L methanol and centrifugation at 12000 r/min for 10 min. The supernatants were analyzed by HPLC for determining drug concentrations.

### 2.6. Animal Experimental Procedures

Eighty-four female C57BL/6 mice (6-month old, body weight 25-33 g, Nrf2 wild genotype) were purchased from the Model Animal Research Centre of Nanjing University (Nanjing, China). Thirty-six Nrf2 knockout (Nrf2 KO) mice (C57BL/6 background) were kindly provided by Dr. Peng Cao (Jiangsu Provincial Academy of Chinese Medicine, Nanjing, China), and the mouse strain was from Johns Hopkins University Medical School (USA). All mice were maintained under a 12 h light/dark cycle at a controlled temperature (25°C) with free access to food and tap water. The Nrf2 wild-type (Nrf2 WT) mice were divided into 7 groups, namely, the aging control group, model control group, QT group (200 mg/kg), Q-PC group (200 mg/kg), and Q-SD groups (50, 100, and 200 mg/kg). The Nrf2 KO mice were divided into 3 groups, namely, the aging control group, model control group, and Q-SD group (200 mg/kg). These doses were defined as the content of pure QT in the phospholipid complex or solid dispersion and were determined by preliminary experiments.

Animals were treated according to the following procedures: (1) For the aging control mice of both Nrf2 WT and Nrf2 KO, they were fed normal diet during months 1-9. (2) For the model control mice of both Nrf2 WT and Nrf2 KO, they were fed with normal diet during months 1-3, then high-fat diet, received the intake of hydroquinone dissolved in the drinking water (0.8%) during months 4-6, and were intragastric administrated with 0.5% CMC-Na suspension daily during months 7-9. (3) For the treatment mice of both Nrf2 WT and Nrf2 KO, they were fed normal diet during months 1-3, then high-fat diet, received the intake of hydroquinone dissolved in the drinking water (0.8%) during months 4-6, and were intragastric administrated with corresponding drugs (suspended in 0.5% CMC-Na) daily during months 7-9. All experimental procedures were approved by the institutional and local committee on the care and use of animals, and all animals received humane care according to the National Institutes of Health (USA) guidelines.

### 2.7. Transmission Electron Microscopy

Eyeballs of three mice in each group were isolated and fixed immediately in 4% paraformaldehyde for 20 min. The cornea, crystalline lens, and vitreous body were removed from the eye tissues. Wall tissue (2 × 4 mm) was excised from the bilateral area of the optic disc and fixed with glutaral/osmic acid, coated with epoxy resins, and sectioned. After double staining with uranyl acetate and lead citrate, the sections were examined with a transmission electron microscope (Tecnai G2 Spirit Bio TWIN; FEI, Hillsboro, OR, USA), and images were taken. The semiquantitative evaluation of width of the sediment beneath the RPE and the thickness of Bruch's membrane was determined according to the methods reported by Espinosa-Heidmann et al. [18].

### 2.8. Measurements of ROS, MDA, and Antioxidant Enzymes

Blood was collected from the orbital cavity of three mice of each group, and serum was isolated. Meanwhile, the eyeball was extracted and the retina was isolated, and tissue homogenates were prepared via centrifugation at 4°C, 3000 r/min for 10 min. The ROS levels in the serum were determined using Fenton's reaction and Griess reagent chromogenic method, and the ROS levels in the retina tissue were determined using the DCFH-DA method. Levels of MDA in serum and retinal tissues were measured using the thiobarbituric acid method. In addition, activities of SOD, GSH-PX, and CAT in serum and retinal tissues were measured using corresponding enzyme-linked immunosorbent assay kits at the wavelength of 450, 412, and 405 nm, respectively. All the kits above were purchased from the Nanjing Jiancheng Bioengineering Institute (Nanjing, China), and experiments were performed according to the instructions provided by the manufactures.

### 2.9. Real-Time PCR

Total RNA was isolated from the retina and choroid of three mice of each group using a TRIzol reagent (Sigma, St. Louis, MO, USA) following the protocol provided by the manufacturer. Reverse transcription was carried out using kits according to the instructions provided by Thermo Scientific Fisher (USA). Glyceraldehyde phosphate dehydrogenase (GAPDH) was used as the invariant control. Fold changes in the mRNA levels of target genes related to GAPDH were calculated. Experiments were performed in triplicate. The primers of genes (GenScript, Nanjing, China) were as follows: Nrf2: (forward) 5′-AGTGACTCGGAAATGGATGAG-3′, (reverse) 5′-TGTGCTGGCTGTGCGTTAGG-3′; HO-1 (forward) 5′-GCTGGTGATGGCTGCCTTGT-3′, (reverse) 5′-ACTGGGTGCTGCTTGTTGCG-3′; HQO-1 (forward) 5′-ATGTATGACAATGGACCCTTCC-3′, (reverse) 5′-TCCCTTGCAGAGTGTCATGG-3′; GCL (forward) 5′-GAAGTGGATGTGGACACTAGATG-3′, (reverse) 5′-TTGTAGTCAGGATGGTCTGCGATAA-3′; and GAPDH (forward) 5′-ATGACATCAAGACGGTGGTG-3′, (reverse) 5′-CATACCAGGTATGAGCTTG-3′.

### 2.10. Western Blot Analysis

Proteins were extracted from the retina and choroid of three mice of each group using a radio-immunoprecipitation assay buffer containing 150 mM NaCl, 50 mM Tris, 0.1% sodium dodecyl sulphate, 1% Nonidet P-40, and 0.5% deoxycholate supplemented with protease inhibitor phenylmethylsulfonyl fluoride. In examining Nrf2 expression, nuclear proteins and cytoplasmic proteins were separated using a Bioepitope Nuclear and Cytoplasmic Extraction Kit (Bioworld Technology, St. Louis Park, MN, USA) according to the protocol. Proteins (50 *μ*g/well) were separated by SDS-polyacrylamide gel, transferred to a PVDF membrane (Millipore, Burlington, MA, USA), blocked with 5% skim milk in Tris-buffered saline containing 0.1% Tween 20. Target proteins were detected by corresponding primary antibodies and subsequently by horseradish peroxidase-conjugated secondary antibodies. Protein bands were visualized using a chemiluminescence reagent (Millipore, Burlington, MA, USA). Equivalent loading was confirmed using an antibody against GAPDH for total proteins and against Lamin B for nuclear proteins. Representative blots were from three independent experiments. The levels of target protein bands were densitometrically determined using Quantity One 4.4.1. The variation in the density of bands was expressed as fold changes compared to the control in the blot after normalization to GAPDH or Lamin B.

### 2.11. Statistical Analysis

Data were presented as mean ± SEM, and results were analyzed using GraphPad Prism 5 software. The significance of difference was determined by Student's *t*-test for comparison between two groups and one-way ANOVA with post hoc Dunnett's test for comparison between multiple groups. A value of *p* < 0.05 was considered to be statistically significant.

## 3. Results

### 3.1. Q-SD Has Better Physicochemical and Pharmacokinetic Properties Than QT and Q-PC

**Figure 1:**
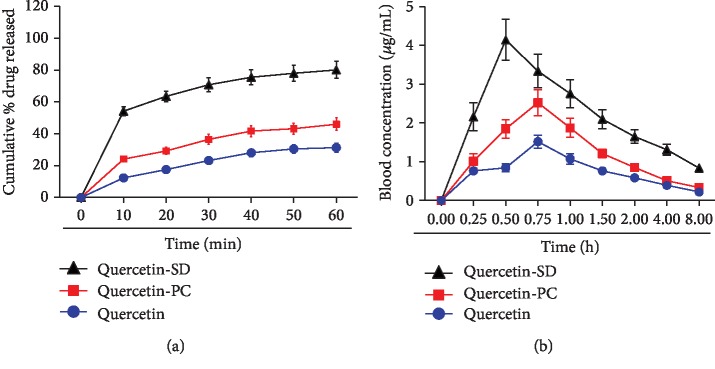
Quercetin-SD has better physicochemical and pharmacokinetic properties than quercetin and quercetin-PC. (a) Determination of cumulative dissolution rates of quercetin, quercetin-PC, and quercetin-SD at indicated time points. (b) Determination of blood concentrations of quercetin, quercetin-PC, and quercetin-SD at indicated time points in rats.

We initially characterized several key physicochemical properties of Q-SD. The results showed that the equilibrium solubility of Q-SD in both water and chloroform was significantly higher than that of QT and Q-PC (Table 1). Determination of the dissolution rate showed that Q-SD had significantly higher cumulative dissolution rates than that of QT and Q-PC at each time point (Figure 1(a)). Next, we determined some key pharmacokinetic parameters of Q-SD in rats and obtained the serum concentration-time curve (Figure 1(b)). The maximum serum concentration of Q-SD was considerably increased to 4.143 *μ*g/mL from 1.517 *μ*g/mL of QT or 2.523 *μ*g/mL of Q-PC. In addition, the area under the curve of Q-SD was remarkably increased to 12.015 *μ*g·h/mL from 5.461 *μ*g·h/mL of QT and 8.074 *μ*g·h/mL of Q-SD. These results collectively demonstrated that Q-SD had better physicochemical and pharmacokinetic properties than QT and Q-PC.

### 3.2. Q-SD More Potently Improves Retina Pathological Changes in Nrf2 WT Model Mice of Dry AMD

**Figure 2:**
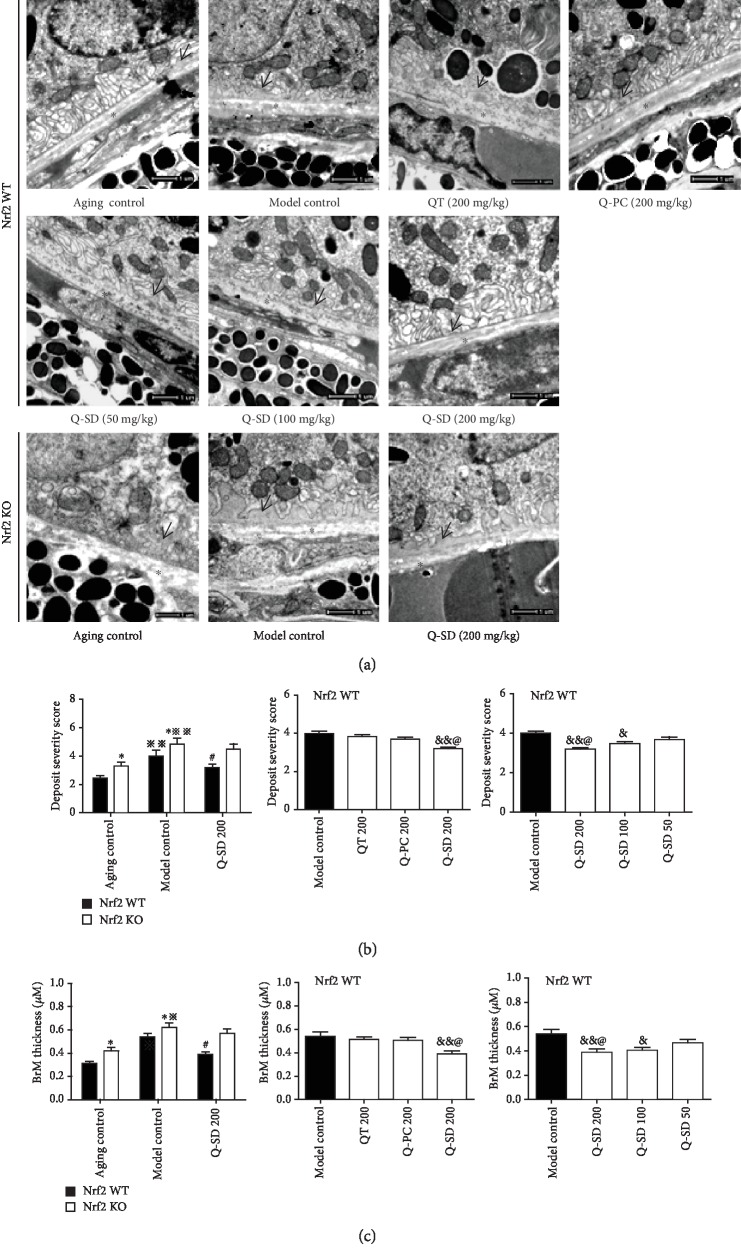
Quercetin-SD more potently improves retina pathological changes in Nrf2 WT mice model of dry AMD. (a) Transmission electron microscopy examination of mouse eye tissues (magnification ×25000). Arrows are used to indicate sediment, and asterisks to indicate Bruch's membrane. (b) Deposit severity score for RPE sediments. (c) Quantification of Bruch's membrane thickness. Significance: ∗*p* < 0.05 Nrf2 WT versus Nrf2 KO in the aging control and model control; ^※^*p* < 0.05, ^※※^*p* < 0.01 model control versus aging control; ^#^*p* < 0.05 Q-SD 200 versus model control; ^&^*p* < 0.05, ^&&^*p* < 0.01 versus model control; ^@^*p* < 0.05 Q-SD 200 versus Q-PC 200 or Q-SD 100 (*n* = 3).

We established the disease model in both Nrf2 WT and Nrf2 KO mice to evaluate the effects of drugs, which reflects typical pathological changes of dry AMD in humans and has been widely used by investigators [18, 19]. Histopathological examinations using transmission electron microscopy showed that there was less spotted sediment and relatively normal Bruch's membrane (BrM) thickness in Nrf2 WT mice of the aging control group, obvious sediment, and thickened BrM in Nrf2 KO mice; massive successive flat sediment and thickened BrM were observed in Nrf2 WT mice of the model control group, but more severe in Nrf2 KO mice (Figure 2(a)). Q-SD at 200 mg/kg distinctly decreased RPE sediment compared to the model control in Nrf2 WT mice, not in Nrf2 KO mice (Figure 2(a)). Consistently, the scoring of sediment severity demonstrated that Q-SD at 200 mg/kg significantly reduced the deposit severity score compared to the QT and Q-PC groups at the same dosage in Nrf2 WT mice and did stronger than that of Q-SD at 100 mg/kg (Figure 2(b)). Furthermore, BrM thickness was significantly reduced by Q-SD at 200 mg/kg in Nrf2 WT mice, not in Nrf2 KO mice and did stronger than that of Q-SD at 100 mg/kg (Figure 2(c)). Collectively, these data indicated that Q-SD more potently improved retina pathological changes in Nrf2 WT, not in Nrf2 KO model mice of dry AMD.

### 3.3. Q-SD Exerts More Potent Antioxidant Effects in Nrf2 WT Model Mice of Dry AMD

**Figure 3:**
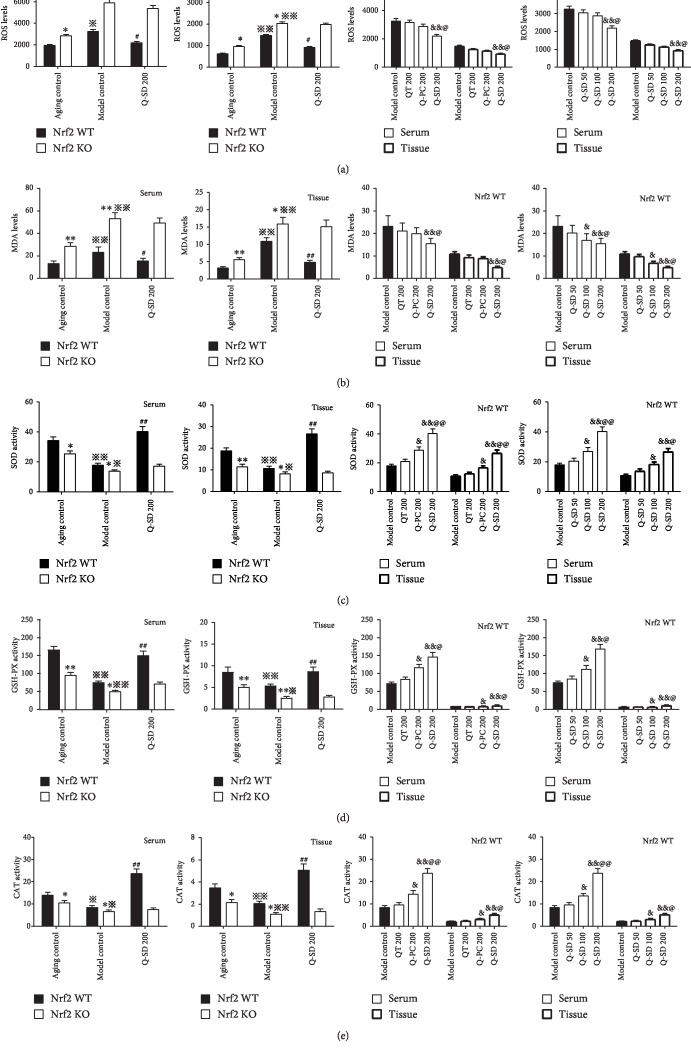
Quercetin-SD exerts more potent antioxidant effects in the Nrf2 WT and Nrf2 KO mice models of dry AMD. (a) Determination of ROS levels in serum and retinal tissues. (b) Determination of MDA levels in serum and retinal tissues. (c) Determination of SOD levels in serum and retinal tissues. (d) Determination of GSH-PX levels in serum and retinal tissues. (e) Determination of CAT levels in serum and retinal tissues. Significance: ∗*p* < 0.05, ∗∗*p* < 0.01 Nrf2 WT versus Nrf2 KO in the aging control and model control; ^※^*p* < 0.05, ^※※^*p* < 0.01 model control versus aging control; ^#^*p* < 0.05, ^##^*p* < 0.01 Q-SD 200 versus model control; ^&^*p* < 0.05, ^&&^*p* < 0.01 versus model control; ^@^*p* < 0.05, ^@@^*p* < 0.01 Q-SD 200 versus Q-PC 200 or Q-SD 100 (*n* = 3).

We speculated that regulation of the redox system could be involved in the effects of Q-SD and thus subsequently examined ROS, MDA, and several antioxidant enzymes in mice. We found that the ROS and MDA levels in both serum and retinal tissues were significantly upregulated in Nrf2 WT and Nrf2 KO mice of the model control group but more obvious in Nrf2 KO mice. Q-SD at 200 mg/kg significantly decreased the ROS and MDA levels, not in Nrf2 KO mice. Q-SD at 200 mg/kg produced the strongest effect on the MDA level compared to that of Q-SD at 100 mg/kg in Nrf2 WT mice (Figures 3(a) and 3(b)). Furthermore, examinations of the three key antioxidant enzymes SOD, GSH-PX, and CAT showed that their activities in serum and retinal tissues were significantly decreased in the model group of Nrf2 WT and more remarkable in Nrf2 KO mice. Treatment with Q-SD at 200 mg/kg significantly increased their activities in serum and retinal tissues in Nrf2 WT mice, not in Nrf2 KO mice. Q-PC at 200 mg/kg and Q-SD at 100 mg/kg also increased their activities in Nrf2 WT mice; as expected, Q-SD at 200 mg/kg yielded the most significant effect (Figures 3(c)–3(e)). Taken together, these results suggested that Q-SD exerted more potent antioxidant effects in the Nrf2 WT model mice of dry AMD.

### 3.4. Activation of Nrf2 Is Associated with the Potent Protective Effects on Retina Injury by Q-SD in Model Mice of Dry AMD

**Figure 4:**
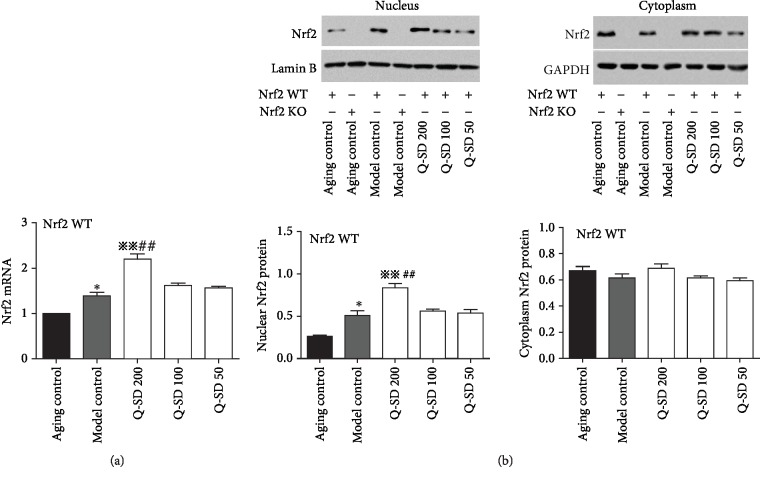
Effects of quercetin-SD on the expression of Nrf2 in Nrf2 WT and Nrf2 KO model mice of dry AMD. (a) Real-time PCR analyses of Nrf2 mRNA expression in retina tissues. (b) Western blot analyses of Nrf2 protein abundance in the nucleus and cytoplasm with quantification. Significance: ∗*p* < 0.05 model control versus aging control; ^※※^*p* < 0.01 Q-SD versus model control; ^##^*p* < 0.01 Q-SD 200 versus Q-SD 100 (*n* = 3).

**Figure 5:**
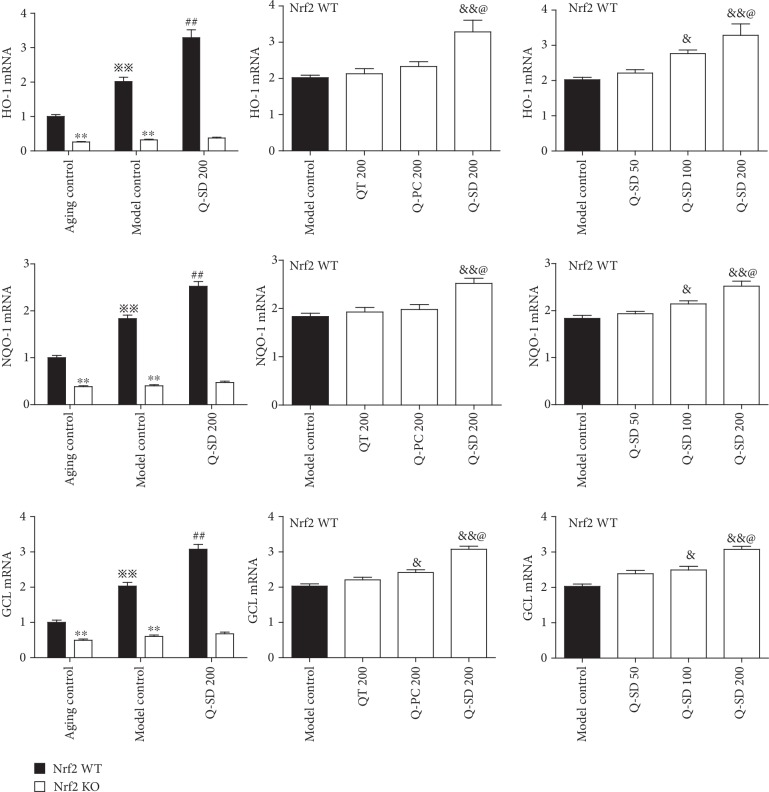
Effects of quercetin-SD on mRNA expression of antioxidant enzymes in Nrf2 WT and Nrf2 KO model mice of dry AMD. Real-time PCR analyses of the mRNA expression of HO-1, NQO-1, and GCL in retinal tissues. Significance: ∗∗*p* < 0.01 Nrf2 WT versus Nrf2 KO in aging control and model control; ^※※^*p* < 0.01 model control versus aging control; ^##^*p* < 0.01 Q-SD 200 versus model control; ^&^*p* < 0.05, ^&&^*p* < 0.01 versus model control; ^@^*p* < 0.05 Q-SD 200 versus Q-PC 200 or Q-SD 100 (*n* = 3).

**Figure 6:**
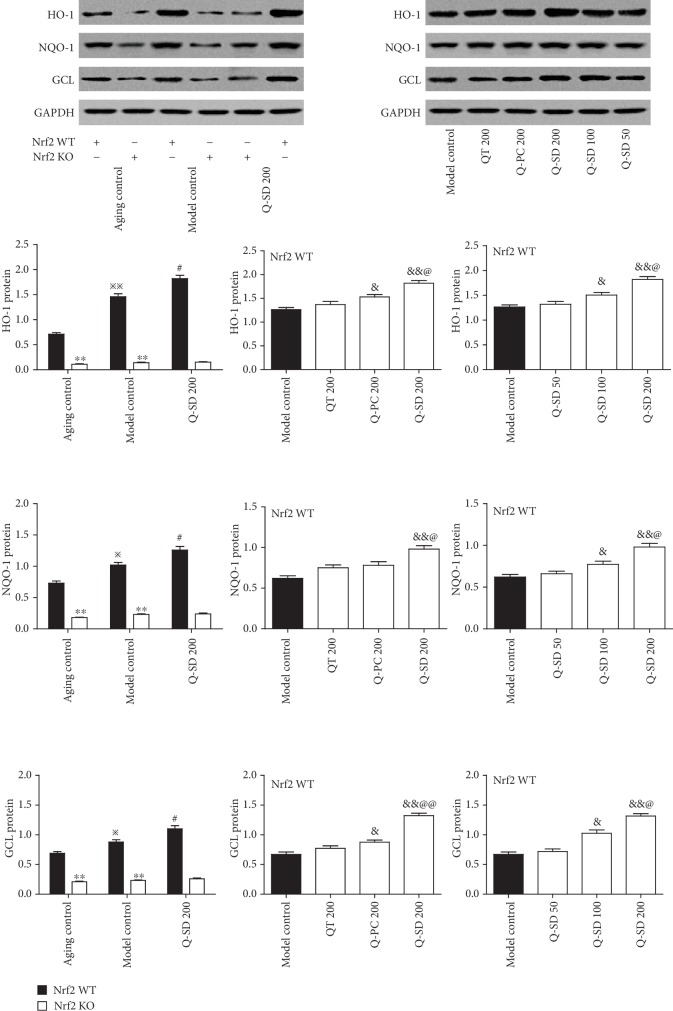
Effects of quercetin-SD on protein expression of antioxidant enzymes in Nrf2 WT and Nrf2 KO model mice of dry AMD. Western blot analyses of the protein expression of HO-1, NQO-1, and GCL in retina tissues with quantification. Significance: ∗∗*p* < 0.01 Nrf2 WT versus Nrf2 KO in the aging control and model control; ^※^*p* < 0.05, ^※※^*p* < 0.01 model control versus aging control; ^#^*p* < 0.05 Q-SD 200 versus model control; ^&^*p* < 0.05, ^&&^*p* < 0.01 versus model control; ^@^*p* < 0.05, ^@@^*p* < 0.01 Q-SD 200 versus Q-PC 200 or Q-SD 100 (*n* = 3).

We further explored the underlying mechanism for Q-SD effects focusing on Nrf2 signaling pathway. We observed that Nrf2 mRNA was markedly increased in retinal and choroid tissues in Nrf2 WT and Nrf2 KO mice; Nrf2 mRNA was markedly increased in Nrf2 WT mice of the model group and was nearly undetectable in Nrf2 KO mice. Q-SD at 200 mg/kg significantly increased Nrf2 mRNA level compared to the model group in Nrf2 WT mice, not in Nrf2 KO mice (Figure 4(a)) (Nrf2 KO mice data not presented in the figure). Moreover, the nuclear abundance of Nrf2 was significantly increased in the model group of Nrf2 WT mice, and Q-SD at 200 mg/kg significantly stimulated nuclear translocation of Nrf2 compared to the model group (Figure 4(b)). However, the cytoplasm abundance of Nrf2 in the model group and Q-SD treatment groups in Nrf2 WT mice was not significantly altered (Figure 4(b)). Nrf2 protein expression in neither nucleus nor cytoplasm was undetectable in Nrf2 KO mice (Figure 4(b)). Furthermore, we examined the expression of major target genes of Nrf2, which are critically involved in the antioxidant system. Real-time PCR analyses showed that the transcript levels of HO-1, NQO-1, and GCL were significantly upregulated in Nrf2 WT mice of the model group and that Q-SD at 100 mg/kg and 200 mg/kg significantly increased their mRNA levels compared to the model group. In the model group Nrf2 KO mice, the mRNA expression of HO-1, NQO-1, and GCL was not significantly enhanced compared to that of the aging control group (Figure 5). Meanwhile, in the model group Nrf2 WT mice, the protein expression of HO-1, NQO-1, and GCL was significantly increased compared to the aging control, and Q-SD at 100 mg/kg and 200 mg/kg significantly upregulated the protein expression of HO-1, NQO-1, and GCL. In Nrf2 KO mice, the protein abundance of HO-1, NQO-1, and GCL was remarkably lower than that in Nrf2 WT mice, and there was no significant difference between the aging control and model control in Nrf2 KO mice (Figure 6). Altogether, these data revealed that activation of Nrf2 was associated with the potent protective effects on retina injury by Q-SD in model mice of dry AMD.

## 4. Discussion

Currently, there are no promising therapeutic options for dry AMD in clinical practice. Accumulating evidence indicates that agents that are able to protect the retina against oxidative stress-induced injury can be an important direction for management of dry AMD. An early clinical research termed Age-Related Eye Disease Study (AREDS) showed that pharmacological use of antioxidants, such as *β*-carotene, vitamin C, vitamin E, zinc, and copper, could prevent AMD progression and reduce 25% risk in advanced AMD progression and 19% risk in moderate vision loss within 5 years [20]. *Fructus lycii* is a well-known tonic medicine frequently used for treating aging-related eye diseases in traditional Chinese medicine system and has been demonstrated to possess antioxidative and antiaging effects [21]. Our previous studies showed that Fructus lycii ethanol extract effectively reduced the RPE sediment and Bruch's membrane thickness in mice with experimental AMD, and its major components lutein and zeaxanthin could protect ARPE-19 cells against hydrogen peroxide-induced oxidative injury [19].

It has been well-recognized that many flavonoid compounds have free radical-scavenging and antioxidant functions, including naringenin, quercetin, and apigenin. However, flavonoid compounds have the disadvantages of poor water- and fat-solubility, low absorption, and less bioavailability [22]. Pharmaceutical studies have revealed that formation of phospholipid complex can markedly improve the physicochemical properties of drugs and enhance their absorption and therapeutic effects in vivo [23]. We previously reported that Q-PC had higher solubility, especially the fat-solubility, than that of QT and that Q-PC thereby exhibited stronger protective effects against oxidative-induced damages in ARPE-19 cells [13]. However, we realized that Q-PC had poor dispersity due to the hydrophobicity of phospholipid complexes. Instead, further preparation of phospholipid complex into solid dispersion can improve the solubility and dissolution rate of the drug, leading to increased bioavailability. For instance, omega-3 phospholipid-based solid dispersion of fenofibrate effectively increased the oral drug exposure in rats, suggesting that this formulation should be promising for improving the oral bioavailability of fenofibrate [24]. Many studies have revealed that the hydrophilic excipient addition PVP could convert drugs to an amorphous form rather than a crystalline state and greatly enhance the solubility and dissolution of drugs [24, 25]. Therefore, we used PVP K30 to prepare Q-SD in the present study. Consistently, our current data demonstrated that formation of solid dispersion tremendously increased the water- and fat-solubility and dissolution rate of Q-PC. More importantly, Q-SD had considerably higher blood concentrations than Q-PC in rats, confirming that Q-SD had high bioavailability. Given that flavonoid drugs have similar pharmacokinetic behaviors in rats and mice, we thus established an animal model of dry AMD in mice to examine the potential effects of QT, Q-PC, and Q-SD on retinal injury caused by oxidative stress. We observed that QT and Q-PC did not reduce RPE sediments and BrM thickness, Q-SD significantly did at the same dosage, and that there were dose-dependent responses in the effects of Q-SD. These data clearly supported the claim that formation of solid dispersion considerably enhanced the protective effects of QT on retinal oxidative injury in mice owing to the increased bioavailability.

Nrf2 signaling had been characterized to be a pivotal antioxidant mechanism in mammal cells. Recent evidence indicated that disruption of Nrf2 gene increased the vulnerability of the outer retina to age-related degeneration. Nrf2-deficient mice developed ocular pathology similar to cardinal features of human AMD related to oxidative injury and inflammation, suggesting that Nrf2 signaling and its downstream target genes played important roles in the pathogenesis of AMD [26]. Regulation of Nrf2 signaling could be a potential strategy for intervention of dry AMD. In line with this recognition, our previous studies demonstrated that apigenin exhibited protective effects on ARPE-19 cells against oxidative injury, which were dependent on activation of Nrf2 signaling [27]. Moreover, Hanneken and co-workers reported that QT could induce Nrf2 and related phase II metabolic enzymes and thus protect ARPE-19 cells against hydrogen peroxide-induced injury [28]. Consistent results were also recaptured by our previous studies. Our current studies used the oxidative injury model in mice to validate the *in vivo* effects. However, due to limited number of Nrf2 KO mice, we only evaluated Q-SD at 200 mg/kg in these animals, because we reasoned that Q-SD at this dose could produce significant effects. We observed increased the mRNA expression and nuclear protein abundance of Nrf2 in the Nrf2 WT mice of the model group, which was extremely low in Nrf2 KO mice. Transcription of HO-1, NQO-1, and GCL was all increased in Nrf2 WT mice, not in Nrf2 KO mice; consistently, the protein expression of these three molecules was not significantly increased in the Nrf2 KO mice of the model group. These results also indicated that the oxidative stress stimulated Nrf2 nuclear translocation and initiated the transcription of phase II metabolic enzymes.

We further found that treatment with Q-SD at 100 mg/kg and 200 mg/kg caused significant increase in the transcription and expression of Nrf2 and phase II metabolic enzymes compared to the model group, whereas QT at the same dose did not show significant effects and Q-PC at 200 mg/kg only shows certain effects on the mRNA and protein expression of GCL and protein expression of HO-1 in Nrf2 WT mice. These data indicated that QT exerted its protective effects through activating Nrf2 signaling and also confirmed that formation of solid dispersion considerably increased the bioavailability of QT *in vivo*. Moreover, QT was found to regulate the expression of antioxidant enzymes SOD, GSH-PX, and CAT via a Nrf2 pathway in HepG2 cells [29]. Our current studies demonstrated similar results showing that QT of different dosage forms could restore the serum and tissue activities of SOD, GSH-PX, and CAT and reduced ROS and MDA levels to different extents in Nrf2 WT model mice of dry AMD, and there was effect-concentration responses in Q-SD effects. Under these conditions, Q-SD at a high dose produced the strongest effect. These observations further confirmed that Q-SD had high bioavailability again. The present studies validated the *in vivo* protective effects of QT on retina injury caused by oxidative stress in mice. Next, we will examine the *in vivo* pharmacokinetic properties of Q-PC and Q-SD for further validation and development.

In summary, preparation of solid dispersion significantly improved the solubility and dissolution rate of QT and thereby increased its bioactivity. Q-SD exhibited more potent protective effects on retina oxidative injury in model mice of dry AMD, which were associated with activation of Nrf2 signaling and related antioxidant enzymes. Our studies highlighted that Q-SD could be a safe and effective therapeutic option for AMD.

## Figures and Tables

**Table 1:** Equilibrium solubility of quercetin of different dosage forms (25°C, *n* = 3).

Solvent	Concentration (*μ*g/mL)		
	Quercetin	Quercetin-PC	Quercetin-SD
Water	20.15 ± 1.45	46.81±2.68^∗∗^	235.54±4.73^∗∗^^##^
Chloroform	2.18 ± 0.25	1.35 × 10^3^±29.22^∗∗^	2.3 × 10^3^±102.9^∗∗^^#^

Significance: ^∗∗^*p* < 0.01 versus quercetin; ^#^*p* < 0.05 versus quercetin-PC; ^##^*p* < 0.01 versus quercetin-PC.

## Data Availability

The data used to support the findings of this study are available from the corresponding author upon request.
